# Composition of *Anopheles* species and bionomic characteristics over the peak malaria transmission season in Bandarban, Bangladesh

**DOI:** 10.1186/s12936-023-04614-2

**Published:** 2023-06-06

**Authors:** Hasan Mohammad Al-Amin, Isabella Rodriguez, Ching Swe Phru, Wasif A. Khan, Rashidul Haque, Bernard L. Nahlen, Timothy A. Burton, Mohammad Shafiul Alam, Neil F. Lobo

**Affiliations:** 1 International Centre for Diarrhoeal Disease Research Bangladesh (icddr,b), Dhaka, Bangladesh; 2 Mosquito Control Laboratory, QIMR Berghofer Medical Research Institute, Brisbane, QLD Australia; 3 School of Biological Sciences, University of Queensland, Brisbane, QLD Australia; 4 Eck Institute for Global Health (EIGH), University of Notre Dame, Notre Dame, IN USA

**Keywords:** *Anopheles*, Bionomics, Human landing catch, Light trap, Bangladesh

## Abstract

**Background:**

Joint efforts by government and non-government organizations have helped to reduce malaria in Bangladesh and set the country on a clear path to eventual malaria elimination. However, achieving that goal would be challenging without a comprehensive understanding of vector bionomics.

**Methods:**

Targeted capturing of *Anopheles* mosquitoes over a rainy season, utilizing specific sampling methods, including human landing catches (HLCs), CDC-light traps (CDC-LTs), and pyrethrum spray catches (PSCs) were aimed to characterize entomological drivers of transmission in four sites of Bandarban, Bangladesh.

**Results:**

Molecular characterization of a subset of 4637 mosquitoes has demonstrated the presence of at least 17 species whose capture rates were representative of the rainy season. Species compositions and bionomic traits did not vary between sites with *Anopheles maculatus* having the highest landing rate by HLCs and *Anopheles vagus* having the highest capture rate with CDC-LTs. Interestingly, *Anopheles* species compositions and capture rates varied significantly (p < 0.05) for *An. vagus*, between HLCs and its often-used proxy—CDC-LTs- suggesting impacts on downstream analysis. CDC-LTs capture rates demonstrated differing compositions with indoor and outdoor biting rates. For example, *Anopheles nigerrimus* and *Anopheles nivipes* were more endophagic by HLCs and more exophagic by CDC-LTs. The use of a cow-baited CDC-LT also demonstrated significantly different results when compared to a human-baited CDC-LT considering the high degree of anthropophily in these species. The exception to both zoophily and indoor resting was *An. vagus*, which demonstrated both anthropophily and high resting rates indoors**—**pointing to this species being a possible primary vector at this site.

**Conclusion:**

A diverse *Anopheles* fauna in Bandarban has been confirmed through molecular methods, highlighting the potential impact of sampling techniques. Given the complexity of the local ecosystem, a better understanding of mosquito behaviour and ecology is required to achieve the goal of malaria elimination in Bangladesh.

**Supplementary Information:**

The online version contains supplementary material available at 10.1186/s12936-023-04614-2.

## Background

Although malaria has always been a prevalent cause of death, particularly in the sub-Saharan Africa, the 21st century has witnessed a significant drop in malaria-associated deaths. The 2022 World Malaria Report released by the World Health Organization (WHO) recorded that regions in South-East Asia have continued to see malaria incidence rate fall, from 18 cases per 1000 population in 2000, to 3.2 cases in 2021 [1]. Malaria case rates have gone down, from about 23 million in 2000 to 5.4 million in 2021. Furthermore, the South-East Asia Region only accounts for 2% of malaria cases worldwide. Most of the malaria cases in Bangladesh originate from the southeastern hilly districts, posing a threat to about 14 million people in the country [2]. There have been concerted efforts by both non-Government organizations (NGOs) and government forces to help lead the malaria elimination efforts within the country [1]. Yet, much still needs to be done that focuses on mosquito behaviour and targets specific vectors based on seasonal and local transmission drivers [3].

Malaria is prevalent in 13 out of 64 districts in Bangladesh, particularly in those districts that share a border with India and Myanmar [4]. Cox’s Bazar and Chittagong Hill Tract (CHT) districts, located in the southwestern corner of Bangladesh, report 90% of malaria cases and over 80% of deaths [5]. Within CHT region, three districts, Bandarban, Khagrachhari, and Rangamati, are the sources of most of the malaria cases. Over 80% of cases occur between May to October, hence are known as the peak malaria transmission season, where an increase in rainfall and high levels of humidity correlate to higher rates of transmission [5].

Until 2016, there were 36 species of *Anopheles* mosquitoes reported within Bangladesh [6–9]. From these, four have been considered as principal malaria vectors: *Anopheles baimaii, Anopheles philippinensis, Anopheles sundaicus* (most likely *Anopheles epiroticus*), and *Anopheles minimus*. Other species, such as *Anopheles aconitus, Anopheles annularis*, and *Anopheles vagus* were found to be vectors during outbreaks. However, in recent times the abundance of principal malaria vectors was found very low and seven more *Anopheles* species: *Anopheles maculatus*, *Anopheles karwari*, *Anopheles nigerrimus, Anopheles barbirostris, Anopheles subpictus, Anopheles jeyporiensis* and *Anopheles nivipes* were found to be incriminated in CHT based on circumsporozoite protein (CSP) enzyme-linked immunosorbent assay (ELISA) [6–8]. Vector data in CHT was scanty until 2010. Articles in the past decade reported the dominance of *An. vagus* abundance along with other species which were not considered important. *Anopheles vagus* has been documented as zoophagic (preference for biting animals), exophilic (ecologically independent of humans), and exophagic (outdoor feeding habits). They are especially prevalent in the plain regions within Bangladesh and have been incriminated during epidemics in those regions [10]. Despite their exophilic behaviour, *An. vagus* has been shown to oviposit in a range of breeding sites including artificial containers and in close proximity to human habitats [10–12]. However, previous faunal studies in malaria endemic areas of Bangladesh have mostly demonstrated CDC light trap (CDC-LT) based collection [6–8]. Such sampling method could be biased for certain species because trap-based collections exploit species-specific behaviour [13, 14]. On the other hand, human landing catches are gold standard and most frequently used methods for mosquito collection [15]. While HLCs and CDC-LTs are used for the active mosquitoes, one of the most common methods is pyrethrum spray catches (PSC) to collect resting indoor mosquitoes. Outdoor resting mosquito collections are always difficult due to the variety of resting sites. Combination of resting and active *Anopheles* collections can give a better idea of species diversity in a given area [16].

Since 2008, vector interventions include the use of insecticide-treated nets (ITNs) later changed to deltamethrin-impregnated long-lasting insecticidal nets (LLINs) in 2013. Indoor residual spraying (IRS) using deltamethrin is also being implemented in hotspots during outbreaks [17]. Though LLINs and IRS are highly effective against endophagic and endophilic *Anopheles* vectors, actual local effectiveness is dependent on the presence of these susceptible behaviours [18, 19]. Therefore, temporal monitoring of entomological endpoints is vital in understanding changes in the drivers of transmission, the vectors. The Entomological Surveillance Planning Tool (ESPT) is a decision-support tool for planning question-based entomological surveillance activities designed for the collection of minimal essential indicators to support cost-effective, locally tailored, and evidence-based vector control [20]. Previous studies at this site have focused on the relationship between cultural practices and malaria rates with little information on the *Anopheles* vector-specific bionomic traits that drive malaria in Bangladesh [21–24]. Recent more rigorous vector morphological and molecular identification methodologies have enabled better species-specific evaluations of vector compositions and their associated behaviours [8, 12].

A better understanding of species behaviour and biting patterns is vital for cost-effective and successful intervention strategies [19, 20]. More species-specific strategies can be implemented that focus on regional drivers of transmission. Fulfilling this knowledge gap will also provide evidence that can outline gaps in protection, residual transmission, and a better understanding of how to respond to regional infections or epidemics. Complex species diversity with associated varying bionomic traits can negatively impact control efforts as similar strategies across a large area can lead to costly efforts that might not yield results based on local drivers. Thus, an understanding of species composition and their behaviours can help target important vectors and consequently transmission. Such data can also outline reasons and causes for regional hotspots for malaria transmission. Knowledge of vector bionomic traits utilized in targeting and tailoring vector control interventions, especially in geographic hotspots, is crucial to effectively eliminate malaria. This study utilized an ESPT-based approach with molecular identifications to characterize vector bionomic traits that impact transmission and intervention effectiveness in four villages within Bandarban district [20].

## Methods

### Site description

The study was conducted in four sites under two unions (Kuhalong and Rajbila) in Bandarban district, Bangladesh (Additional file 1: Fig. S1) [25]. The temperature in Bandarban district ranges from 13 °C in winter to 34.6 °C in summer. The rainy season lasts from the end of May until October, with an average of about 400 mm of rain per month (data obtained from Soil Resources Development Institute, SRDI, Bandarban). Sites included in the study were based on high malaria endemicity, elevation, and ecological representativeness (crop cultivation sites including rubber plantations, rice fields, “Jhum” cultivation i.e. slash and burn agricultural practice) (Additional file 1: Fig. S1). The four sites included were (a) Rubber Bagan, (b) Noa Para, (c) Prue Mong U Headman Para, and (d) Jogesh & Chikka Para. Rubber Bagan with a total of 68 households, is surrounded by rubber plantations – ecologically different from other sites based on monoculture. The rubber trees (*Hevea brasiliensis*) have sap collection cups placed on them which collect rainwater and potential larval sites for several *Anopheles* species including *An. vagus* - documented to oviposit in artificial containers in Bandarban [12]. Noa Para is characterized by rice fields and adjacent Jhum cultivation areas, with a water canal at its northern limit. This site has 41 households and two small cattle sheds. The inhabitants of Noa Para are primarily agriculturists, work in the Jhum sites, and are a high-risk population for malaria infection [26]. Prue Mong U Headman Para is at the highest elevation (105 m) and consists of 55 households - all on top of hills. The lowland areas surrounding these hills consist of rice fields with dams retaining water. Jogesh & Chikka Para consists of several adjacent house clusters (villages) and is the only site situated in Rajbila union. Altogether this site has 89 households with rice fields positioned to the east.

### Sampling methods

Entomological sampling was conducted monthly from May to October 2018, encompassing the peak malaria transmission session. Deviations from the standard collection routines outlined below were based on the number of collections at each site and were factored into the analysis.

*Human landing catch *(*HLC*): Paired indoor and outdoor human landing catches (HLC) were conducted in one sentinel house for two nights per site per month for a total of 48 trapping nights. Collections started at 1800 h and ended at 0600 h.

*CDC-light traps* (*CDC-LT*): Collections were conducted in 10 randomly selected sentinel houses (four indoor collections + four outdoor collections + two animal shed collections) for three days per month per site (for a total of 720 trapping nights). Collections started at 1800 h and ended at 0600 h. Traps were deployed next to a person sleeping under a net for indoor collections, in the outside yard of another house for outdoor collections, and inside an animal shed with cattle and/or goats for animal shed collections. Indoor and outdoor sampling houses did not overlap each other.

*Pyrethrum spray catches* (*PSCs*): Collections were performed at randomly selected houses (excluding HLC and CDC-LT sentinel houses), for three days per week per site. No repeated collection was made within a month. All houses chosen represented typical local household construction materials - wood and bamboo. Collections were made between 0700 and 1000 h.

*Sample processing*: Following collection, all *Anopheles* mosquitoes were morphologically identified to species following standard taxonomic keys and placed in 1.5 ml Eppendorf tubes with desiccant, and labelled with individual mosquito codes [11, 27, 28]. Each code contained morphological identification, trap type, collection location (indoor/outdoor), house code, hour of collection (for HLC and PSCs), and date.

A subset of morphologically identified mosquitoes was sequenced at the ribosomal DNA internal transcribed spacer region 2 (ITS2) (n = 588) and/or cytochrome oxidase subunit 1 (*CO1*) loci (n = 74) towards species determination [29–33]. Samples sequenced were randomly chosen across trapping method and site. In addition to these randomly chosen samples, all specimens with uncertain morphological identifications were also sequenced. Samples were first sequenced at the ITS2 loci, and then a subset of samples with successful ITS2 sequences was also sequenced at the *CO1* loci. Molecular identification was conducted blind to morphological identity to prevent any bias in the analysis. Final species confirmation required high sequence identity (thresholds of 96% for ITS2, and 94% for *CO1*) to sequences in multiple databases – NCBI nr and BOLD [31, 34]. *CO1* and ITS2 database comparisons for each sample were paired to determine species when either *CO1* or ITS2 alone did not produce significant results to voucher sequences. Consensus sequences were manually inspected for insertions, deletions, and repeat regions to ensure these sequence differences did not inflate divergence and decrease identity scores. Consensus sequences of each sequence group were compared (BLASTn) to the NCBI nr and BOLD databases to identify species [34].

### Data analysis

Negative binomial generalized linear models (GLM) were generated to analyse the differences of capture rates in terms of incidence rate ratio (IRR) between CDC-LT and HLC for the top seven *Anopheles* species. These models were used to investigate disparities in host preference among mosquitoes captured by CDC-LT and to explore hourly variations in overall *Anopheles* mosquito activity. Models for trap performance and indoor/outdoor behaviour included trap type and location, and their interaction as predictors. Host preference models used CDC-LT data alone and included the host nearest the trap (human compared to animal) as a predictor. Hourly differences in *Anopheles* landing behaviour were modelled by comparing the behaviour at each hour to all other hours to identify relative periods of increased or decreased activity. All models included the day of collection as a numeric predictor to account for seasonal changes in mosquito behaviour. Data cleanup and statistical analysis was performed in R version 4.1.2. Negative binomial models were generated using the ‘glm.nb’ function from the ‘MASS’ package [35, 36].

## Results

*Anopheles* mosquitoes (n = 4637) were sampled from Bandarban, Bangladesh, from May through October (the rainy season) of 2018. Multiple sampling methods including HLCs, CDC-LTs, and PSCs were utilized to understand behaviour-based species compositions. For active mosquitoes, HLCs and CDC-LTs were used to understand spatial and temporal biting rates, and zoophily versus anthropophily, while for resting mosquitoes, PSCs were used to estimate indoor resting densities.

### Molecular species determination

Molecular identification of a subset of samples (n = 588) resulted in 17 species. These species included *Anopheles culicifacies, An. minimus, Anopheles montanus, An. nigerrimus, Anopheles tessellatus* and *Anopheles umbrosus* were not identified molecularly. Overall 64.5% of specimens were morphologically identified correctly (ranging from 100 to 0% based on the species). Species with morphological identifications that were more than 85% correct based on molecular results included *An. baimaii*, species in the *An. barbirostris* complex, *An. jeyporensis, An. maculatus, An. subpictus* and *An. vagus*. Sequencing demonstrated the presence of 17 species (Table 1). All species, with the exception of *An. splendidus* and *An. philippinensis* had both ITS2 and CO1 samples. *Anopheles baimaii*, part of the *Anopheles dirus* complex, was confirmed using PCR [37], while both *Anopheles campestris* and *Anopheles dissidens* (part of the *An. barbirostris* complex) were confirmed by comparing sequences generated to the PCR sequences used to for diagnostic assays [29, 38]. Two types of *An. subpictus* sequence groups were detected with each ITS2 and CO1 sequence pair having matching sequences within each group. One sequence pair was determined to be Form A while the other was determined to be Form B using sequence comparisons to specimens (MW078486, MW078485, MW078484) from each form [39–41].

**Table 1:** Morphological versus molecular identifications

Molecular species	Morphological Identification correct (#)	ITS2			CO1			
		NCBI Coverage (%)	NCBI E-value	NCBI % Identity (%)	NCBI coverage (%)	NCBI E-value	NCBI %ID	BOLD % ID
An. baimaii*	100% (n = 54)	97	0	99.59	99	0	98.91	99.84
An. campestris*	90% (n = 10)	98	0	97.75	97	0	97.51	97.5
An. dissidens*		99	0	100	98	0	99.68	100
*An. jamesii*	63% (n = 16)	98	0	98.89	99	0	99.37	100
*An. jeyporensis* ^*@*^	90% (n = 21)	99	0	99.72	97	0	99.39	99.84
*An. karwari* ^*@*^	78% (n = 18)	100	0	99.77	99	0	91.61	99.67
*An. kochi* ^*@*^	59% (n = 17)	99	0	100	97	0	99.51	99.84
*An. maculatus* ^*@*^	86% (n = 22)	99	0	100	100	0	99.69	100
*An. nivipes* ^*@*^	35% (n = 43)	95	0	98.59	99	0	92.13	100
*An. peditaeniatus* ^*@*^	34% (n = 42)	100	0	100.00	97	0	99.84	100
*An. philippinensis* ^*@*^	0% (n = 3)	99	0	99.55	–	–	–	–
*An. sawadwongporni*	0% (n = 6)	99	0	99.30	99	0	93.68	100
*An. splendidus* ^*@*^	0% (n = 8)	99	0	99.78	–	–	–	–
*An. subpictus* Form A ^*, @^	100% (n = 5)	100	0	100	99	0	99.53	99.84
*An. subpictus* Form B ^*, @^	100% (n = 1)	100	0	100.00	99	0	99.40	99.21
*An. vagus* ^*, @^	92% (n = 13)	99	0	100	99	0	97.90	99.56
*An. varuna* ^@^	53% (n = 23)	100	0	98.92	98	0	98.72	99.18

Seventeen species were identified using ITS2 and CO1 sequences. The percentage with matching morphological identifications and the number of specimens sequenced are noted. Two species did not produce viable CO1 sequences (−). Species identities labelled with * were confirmed using diagnostic PCRs. Species with sequences that matched voucher specimens are marked with ^@^

**Fig. 1:**
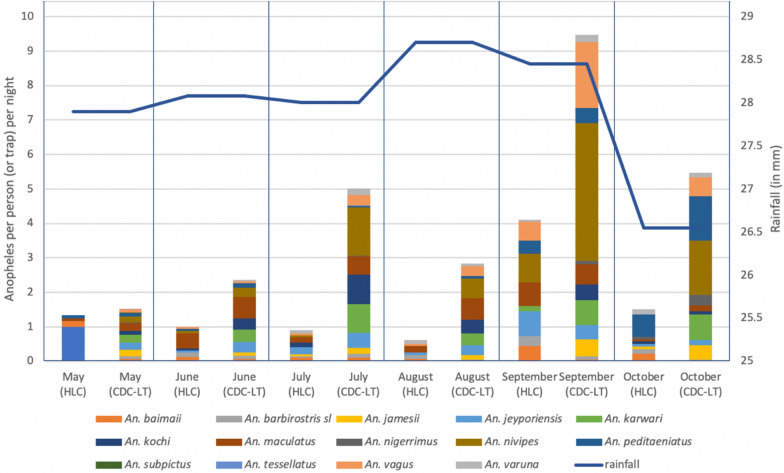
CDC-LT and HLC species-specific temporal capture rates. Though trends remained the same with peak populations following peak rainfall, species-specific compositions and capture rates varied by the sampling device used

*Seasonality*: Both CDC-LTs and HLCs were used to understand species compositions and seasonality during the rainy season - May to October. Catching rates reflected rainfall and sampling method with September having the highest *Anopheles* densities, with a large proportion consisting of both *An. nivipies* followed by *An. vagus*. *Anopheles nivipes* consistently contributed to a large portion of the monthly *Anopheles* catches (Fig. 1).

**Fig. 2:**
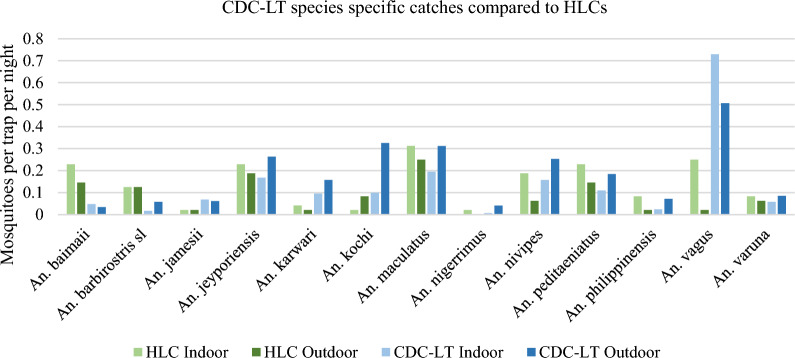
Comparison of HLC and CDC-LT catches. Each sampling device capture rates varied for each species

*Trap comparisons*: Species-specific trap comparisons were analysed from the 14 species identified morphologically. Unidentified specimens were not included in the analysis. CDC-LTs, HLCs, and PSCs had independent species-specific sampling rates when standardized to mosquitoes per trap per night CDC-LTs captured all 14 species while HLCs captured 13, and PSCs captured ten species. *Anopheles vagus*, *Anopheles kochi*, and *An. maculatus* were caught most outdoors by both CDC-LT and HLC trapping method while *An. vagus* and *An. maculatus* were the predominant species caught indoors by these methods. CDC-LT, when compared to HLCs demonstrated differing results with respect to both species, species-specific densities as well as based on location (inside and outside). For example, HLC sampling rates inside and outside were 0.15 and 0.02 *An. vagus*/person/night while CDC-LT sampling rates were 0.72 and 0.79 *An. vagus*/trap/night respectively—with CDC LTs capturing 4.8 × more specimens inside and 24.5 × more outdoors. This increased sampling rate in CDC LTs was not consistent across species. CDC-LTs using animals as bait also captured the most of all species—with the exception of *An. vagus. Anopheles nivipes* was, by far the most prevalent in animal-baited CDC-LTs (5.71/trap/night). PSCs, indicative of resting behaviour, captured 10 species with *An. vagus* being the most prevalent (Additional file 1: Table S1) (Fig. 2).

Negative binomial models of mosquito prevalence based on trap type (HLC versus CDC-LT) and location (indoor versus outdoor) were generated for each of the seven most prevalent morphologically identified *Anopheles* species by total collection. *Anopheles vagus* had significantly higher collection by CDC-LT (IRR, CDC-LT VS HLC: 3.74, p = 0.042), and nearly significantly lower outdoor HLC compared to indoor HLC (IRR: 0.11, p = 0.061). *Anopheles nivipes* had significantly higher outdoor collection by CDC-LT (IRR, outdoor CDC-LT vs. indoor CDC-LT: 6.02, p = 0.029). Models of the other five species (*An. karwari, An. maculatus, An. kochi, Anopheles peditaeniatus, An. jeyporiensis)* did not show significant trap differences.

### Bionomic traits—biting behaviours indoor versus outdoor (based on HLCs and CDC-LT)

**Fig. 3:**
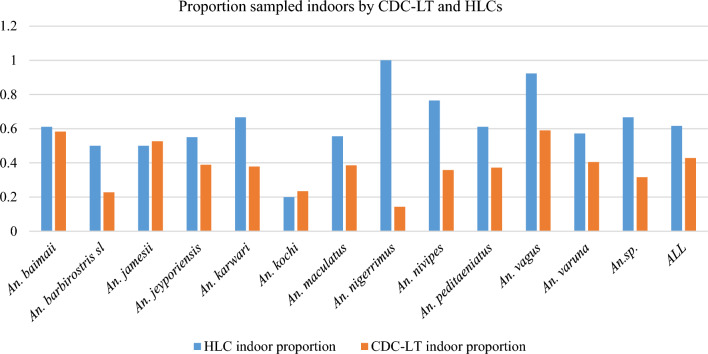
The proportion of each species that were captured indoors using CDC-LTs and HLCs. Species and location-specific capture rates were different based on sampling type. Interestingly, HLCs suggested endophagy for *An. nigerrimus* while CDC-LTs suggested exophagy

Indoor biting rates calculated were dependent on species and sampling method. Overall, in Bandarban, HLCs had higher catches indoors while CDC-LTs captured more outdoors (Fig. 3). *Anopheles nigerrimus* and *An. nivipes* were more endophagic by HLCs and more exophagic by CDC-LTs (if being used as a proxy of HLC).

### Bionomic traits—biting behaviours time of biting (based on HLCs)

**Fig. 4:**
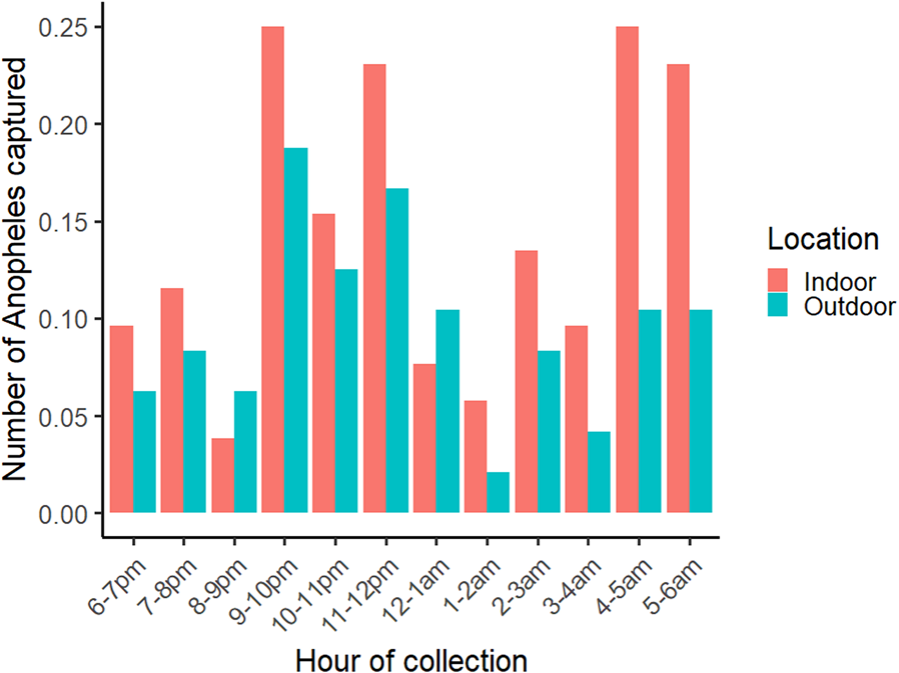
Hourly *Anopheles* mosquito activity as measured by HLCs. Hourly behaviour is displayed on the x-axis from 1800 to 0600 h, with colors representing the location of collection (indoor vs. outdoor)

To determine the biting time and overall mosquito activity during the night, HLCs were conducted inside and outside houses. Results indicate that biting activity is generally low and slightly variable throughout the night, with higher periods of biting activity appearing to overlap with human activity—with a late evening to midnight peak and an early morning peak. The number of specimens sampled by hour was usually too low to determine species-specific biting behaviour over the course of the night (Fig. 4).

### Bionomic traits—anthropophily versus zoophily (CDC-LT versus CDC-animal traps)

Species-specific host preferences (animals versus humans) were determined based on the proportion of *Anopheles* approaching humans compared to cows. Overall, *An. vagus* demonstrated the most anthropophilic behaviour.

**Fig. 5:**
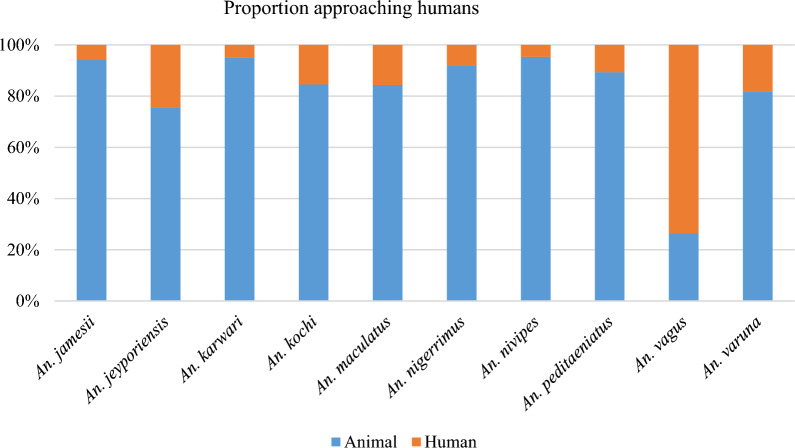
The proportion of each species approaching animals (CDC-LT animals) versus humans (inside and outside CDC-LTs).

When evaluating anthropophily versus zoophily (using human versus cow-baited CDC-LTs), of the 11 species analysed, almost all demonstrated zoophily with a smaller proportion of anthropophily. Though *An. nivipes* was the most common *Anopheles* sampled, only one species, *An. vagus*, was predominantly anthropophilic (Fig. 5). Negative binomial models of light trap host preference, comparing the CDC-LT activity in light traps located in or outside human dwellings compared to those located near animals. Incidence rate ratios (IRR) are reported, with IRR greater than 1 indicating a preference to human-oriented light traps (Table 2).

**Table 2:** CDC-LT-based host preference indicates that *An. vagus* to be primarily anthropophilic

Species	Human-oriented trap (IRR vs. animal baited)
*An. nivipes*	(IRR: 0.047, p < 0.001)
*An. vagus*	(IRR: 2.84, p = 0.0075)
*An. karwari*	(IRR: 0.054, p < 0.001)
*An. maculatus*	(IRR: 0.19, p < 0.001)
*An. kochi*	(IRR: 0.18, p < 0.001)
*An. peditaeniatus*	(IRR: 0.10, p < 0.001)
*An. jeyporiensis*	(IRR: 0.32, p < 0.001)

Incidence rate ratios (IRR) greater than 1 indicate anthropophily

### Bionomic traits—primarily anthropophilic. Incidence raindoor resting (based on PSCs)

PSCs were used to understand indoor resting *Anopheles* species. Of the 10 species found resting indoors, only one was found in significant numbers—*An. vagus* (n = 1027) (Additional file 1: Table S1). When looking at the number of mosquitoes that rest indoors on walls relative to the number that approach houses (by CDC-LT and HLC) it was seen that 18 × more *An. vagus* rest indoors (2.52 /structure/night) than land on humans (0.14 /person/night) and 4x more rest indoors than are caught in human baited-CDC-LTs inside and outside households (0.62 / trap per night). Approximate 81.9% of *An. vagus* resting inside structures were morphologically scored as blood-fed.

## Discussion

Towards understanding baseline entomological bionomic traits that impact both transmission and intervention efficacy, entomological surveys were conducted in four sites of Bandarban, Bangladesh. The high diversity of species found in these sites concurs prior studies [7, 8, 12]. The limitations of morphological identification with regards to the proportion identified correctly have also been reported in other studies [29, 30, 32, 33]. Molecular identification in combination with morphological identification, based on the species present at the site along with historical data may produce the most representative data when evaluating the presence of species or species diversity. The high diversity of species (at least 17) in this study represents multiple ecological niches and bionomic traits that may complicate specific intervention strategies since interventions take advantage of specific susceptible *Anopheles* bionomic traits. Confirmed by molecular identification this study added three new country records of species, namely *An. campestris*, *An. dissidens* and *Anopheles sawadwongporni*.

Though the four sites were selected to represent multiple ecotypes, there was no relationship seen between mosquito densities and land use or ecotype (by site). A comparison of the species-specific trapping efficacies by each sampling methods demonstrated the intrinsic biases of each sampling method which may contribute to the final data output. Sampling methods take advantage of specific mosquito behaviours resulting in trap-based differences in both species and densities [14]. HLCs have been considered the ‘gold standard’ based on them targeting anthropophagic and human-host-seeking mosquitoes [42]. Since HLCs may have ethical issues, CDC-LTs are often used as a proxy for determining important entomological endpoints such as the human biting rate [43]. Data presented here suggest that based on how the sampling methods function at a specific site, CDC-LTs may not be an appropriate proxy for HLCs—since variations in capture rates may impact analysis outputs. If used as a proxy for HLCs, CDC-LT-based conversion factors may have to be determined for both species and location (indoor versus outdoor) since divergent capture rates were seen in this study for both species and location. PSCs, that catch indoor resting mosquitoes, caught the fewest densities and species—with the exception of *An. vagus.* Here, the compositions and densities of species captured resting indoors- relative to those seen in HLCs and CDC-LT captures, points to the importance of selecting appropriate sampling methods based on objective (or question being asked) of the entomological sampling.

Though species-specific, CDC-LT-based capture rates when using a human versus animal bait, demonstrated zoophily. Though the larger biomass of the cow (when compared to a human) may have contributed to the higher attraction to the animal seen, previous reports have also documented zoophily [44]. The higher number of mosquitoes being attracted to animals suggests the possibility of utilizing animals using endectocides. These are the drugs applied to animals which will kill biting mosquitoes [45]. Indoor versus outdoor capture rates were also impacted by the sampling method. The higher indoor anthropophagy seen with HLCs suggests that LLINs and IRS may be useful interventions at this site [3, 18]. The differences seen here with the two sampling devices may outline differences in endo- and exophagy (HLCs) versus endo- and exophily (CDC-LTs). Here the larger proportion of *An. nigerrimus* and *An. nivipes* seen indoors with HLCs versus CDC-LTs, possibly indicate higher anthropophagy or prefer feeding on humans (HLCs) versus anthropophily or prefer being around humans than other hosts (CDC-LTs) (Fig. 3).

*Anopheles* biting over the entire night with peaks that coincided with human activity (late evening and early morning) demonstrate that exposure may happen both indoors and outdoors throughout the night. All species sampled were found both indoors and outdoors. These biting behaviours suggest LLINs would be effective against panmictic populations of each species that bite both indoors and outdoors – but also point to gaps in protection – spaces and times when they may not be as effective [3].

Results demonstrate that the indoor resting behaviours of *An. vagus* was in contrast to those of all the other species seen. In this study, *An. vagus* is highly endophilic with many more resting on walls than were found approaching structures (by HLCs or CDC-LTs). This in combination with its highly anthropophilic nature suggests that *An. vagus* is a primary vector at this site, although historically it was considered a secondary and epidemic vector [10]. Previous studies documented high abundance of *An. vagus* with *Plasmodium* infections from adjacent study sites [6–8]. Interestingly, this species was found to be resistant to pyrethroid insecticides indicating possible selection based on both feeding behaviours and indoor resting resulting in more species-specific contact with indoor insecticides [46].

The limitations of this study include the timeline of collections—only May to October, encompassing the rainy season, omitting the drier seasons where other species may be present and contribute to transmission. The limited number of households included in the study was a result of operational research constraints. However, despite the restricted sampling frame, a significant number of *Anopheles* mosquitoes were captured. The limited molecular sample (n = 588 of 4637 female *Anopheles* specimens) allows for the possibility of there being additional species present at this site.

## Conclusion

This study presents significant insights into the faunal composition and bionomic characteristics of *Anopheles* species in the high malaria endemic district Bandarban, Bangladesh. Findings in this study highlight the importance of careful consideration and appropriate selection of sampling techniques, as species composition, capture rates, and bionomic traits varied between collection methods. Notably, *An. vagus* exhibited high levels of anthropophily, abundance, and endophily, indicating its role as the primary malaria vector in Bangladesh. These findings have important implications for the design of effective control measures and the prevention of malaria transmission in Bangladesh.

## Supplementary Information

**Additional file 1: Figure S1.** A. Map of Bangladesh with the malaria endemic regions labeled in yellow. Bandarban District, with the highest malaria is colored orange with the study site outlined in blue. B. The study sites (1) Noa Para, (2) Rubber Bagan, (3) Prue Mong U Headman Para, and (4) Jogesh and Chikka Para. The map was published in an accompanying article (https://doi.org/10.1186/s12936-022-04375-4) which was a part of the same project. **Table S1.** Trap-based sampling comparisons. Trapping method, species, and location impacted sampling rates.

## Data Availability

The datasets supporting the conclusions of this article are available from the corresponding author upon reasonable request.

## Abbreviations

WHO: World Health Organization
NGO: Non-Government Organization
CHT: Chittagong Hill Tracts
CSP: Circumsporozoite protein
ELISA: Enzyme-linked immunosorbent assay
ITN: Insecticide treated nets
LLIN: Long-lasting insecticidal net
IRS: Indoor residual spray
ESPT: Entomological Surveillance Planning Tool
SRDI: Soil Resources Development Institute
HLC: Human landing catch
CDC: Center for Disease Control and Prevention
LT: Light trap
PSC: Pyrethrum spray catch
ITS: Internal transcribed spacer
COI: Cytochrome oxidase subunit
NCBI nr: Non-Redundant Database by National Center for Biotechnology Information
BOLD: Barcode of life data system
BLAST: Basic local alignment search tool
GLM: Generalized linear model
IRR: Incidence rate ratio
