# Supplementary material for: Composition of Anopheles species and bionomic characteristics over the peak malaria transmission season in Bandarban, Bangladesh
Source: Malar J. 2023 Jun 6;22:176. doi: 10.1186/s12936-023-04614-2 (PMC10245547; doi:10.1186/s12936-023-04614-2)
Supplement: Supplementary file 1 — Additional file 1: Figure S1. A. Map of Bangladesh with the malaria endemic regions labeled in yellow. Bandarban District, with the highest malaria is colored orange with the study site outlined in blue. B. The study sites (1) Noa Para, (2) Rubber Bagan, (3) Prue Mong U Headman Para, and (4) Jogesh and Chikka Para. The map was published in an accompanying article (https://doi.org/10.1186/s12936-022-04375-4) which was a part of the same project. Table S1. Trap-based sampling comparisons. Trapping method, species, and location impacted sampling rates. [file 12936_2023_4614_MOESM1_ESM.docx]

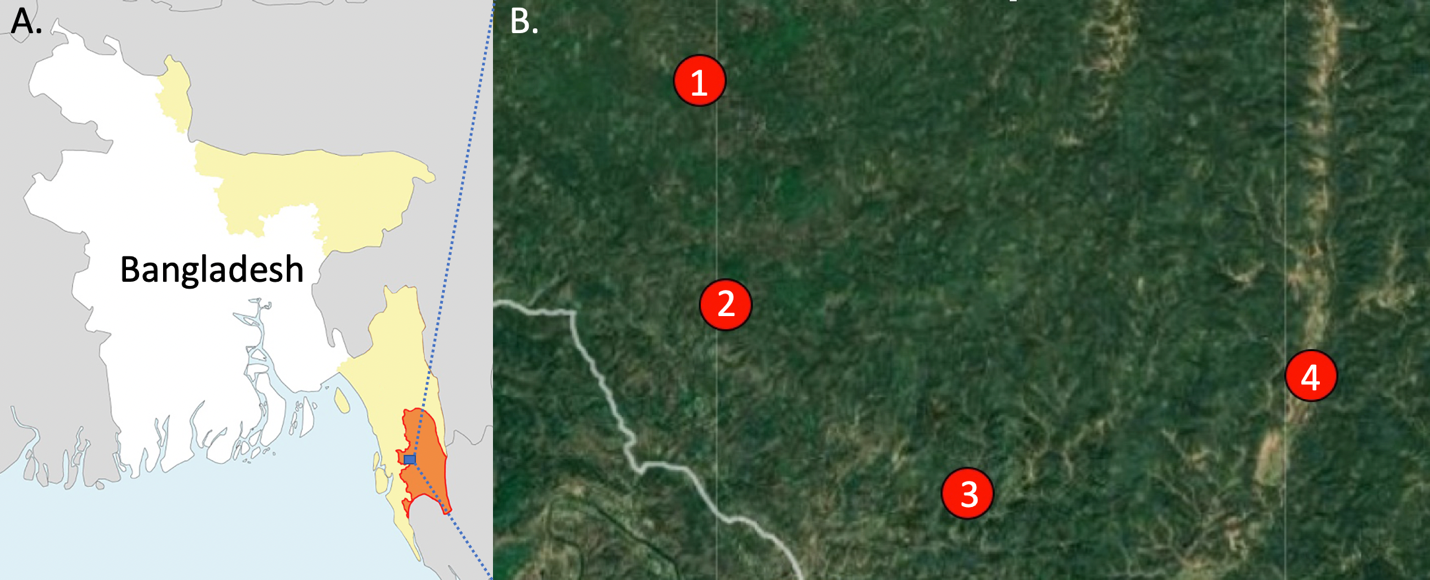


**Figure 1. A.** Map of Bangladesh with the malaria endemic regions labeled in yellow. Bandarban District, with the highest malaria is colored orange with the study site outlined in blue. **B.** The study sites 1) Noa Para, 2) 1,2,3 Rubber Bagan, 3) Prue Mong U Headman Para, and 4) Jogesh and Chikka Para. The map was published in an accompanying article (https://doi.org/10.1186/s12936-022-04375-4) which was a part of the same project.

**Table 1.** Trap-based sampling comparisons. Trapping method, species, and location impacted sampling rates.

| ***Anopheles* Species** | **HLC (Indoor)** | | **HLC (Outdoor)** | | **CDC-LT (Indoor)** | | **CDC-LT (outdoor)** | | **CDC-LT (Animal)** | | **PSC** | |
| --- | --- | --- | --- | --- | --- | --- | --- | --- | --- | --- | --- | --- |
|  | **Total caught** | **/trap /night** | **Total caught** | **/trap /night** | **Total caught** | **/trap /night** | **Total caught** | **/trap /night** | **Total caught** | **/trap /night** | **Total caught** | **/trap /night** |
| *An. baimaii* | 13 | 0.27 | 7 | 0.15 | 14 | 0.05 | 10 | 0.03 | 17 | 0.12 |  |  |
| *An. barbirostris* | 3 | 0.06 | 6 | 0.13 | 5 | 0.02 | 16 | 0.05 | 23 | 0.16 | 2 | 0.00 |
| *An. jamesii* | 1 | 0.02 | 1 | 0.02 | 16 | 0.05 | 16 | 0.05 | 115 | 0.82 |  |  |
| *An. jeyporiensis* | 11 | 0.23 | 9 | 0.19 | 44 | 0.15 | 66 | 0.23 | 66 | 0.47 | 1 | 0.00 |
| *An. karwari* | 2 | 0.04 | 1 | 0.02 | 25 | 0.09 | 41 | 0.14 | 283 | 2.02 |  |  |
| *An. kochi* |  |  | 4 | 0.08 | 29 | 0.10 | 95 | 0.33 | 158 | 1.13 | 3 | 0.01 |
| *An. maculatus* | 14 | 0.29 | 11 | 0.23 | 56 | 0.19 | 89 | 0.30 | 171 | 1.22 | 1 | 0.00 |
| *An. nigerrimus* | 1 | 0.02 |  |  | 2 | 0.01 | 12 | 0.04 | 39 | 0.28 | 8 | 0.02 |
| *An. nivipes* | 12 | 0.25 | 4 | 0.08 | 53 | 0.18 | 93 | 0.32 | 800 | 5.71 |  |  |
| *An. peditaeniatus* | 10 | 0.21 | 6 | 0.13 | 28 | 0.10 | 52 | 0.18 | 152 | 1.09 | 7 | 0.02 |
| *An. subpictus* |  |  |  |  | 1 | 0.00 | 1 | 0.00 | 2 | 0.01 | 4 | 0.01 |
| *An. tesselatus* | 5 | 0.10 |  |  | 2 | 0.01 | 2 | 0.01 | 13 | 0.09 | 34 | 0.08 |
| *An. vagus* | 7 | 0.15 | 1 | 0.02 | 211 | 0.72 | 144 | 0.49 | 22 | 0.16 | 1027 | 2.45 |
| *An. varuna* | 4 | 0.08 | 3 | 0.06 | 24 | 0.08 | 32 | 0.11 | 48 | 0.34 | 12 | 0.03 |
| **Total** | 83 | 1.72 | 53 | 1.11 | 510 | 1.75 | 669 | 2.28 | 1909 | 13.62 | 1099 | 2.62 |
